# Berberine and celecoxib synergistically suppress non-small cell lung cancer with EGFR/AKT/COX-2-related signaling: network pharmacology, molecular docking and experimental validation

**DOI:** 10.3389/fphar.2026.1930125

**Published:** 2026-09-29

**Authors:** Qiang Wang, Chengyu Bian, Xinyuan She, Haonan Du, Minhe Wang, Yu Zheng, Lei Cao, Chenghao Fu, Yuheng Wang

**Affiliations:** 1 Department of Thoracic Surgery, Taizhou Fourth People’s Hospital, Taizhou, Jiangsu, China; 2 Department of Thoracic Surgery, Shanghai Pulmonary Hospital, School of Medicine, Tongji University, Shanghai, China; 3 Department of Pathology, Jiangsu Province (Suqian) Hospital, Suqian, Jiangsu, China; 4 Department of Thoracic Surgery, The First Affiliated Hospital of Xi’an Jiaotong University, Xi’an, Shaanxi, China; 5 Department of Thoracic Surgery, Jiangsu Province People’s Hospital and The First Affiliated Hospital of Nanjing Medical University, Nanjing, Jiangsu, China; 6 Department of Oncology, Jiangsu Province (Suqian) Hospital, Suqian, Jiangsu, China; 7 Department of Thoracic Surgery, Suzhou Municipal Hospital and The Affiliated Suzhou Hospital of Nanjing Medical University, Suzhou, Jiangsu, China

**Keywords:** berberine, celecoxib, drug synergy, EGFR/AKT/COX-2 signaling, non-small cell lung cancer (NSCLC)

## Abstract

**Background:**

Non-small cell lung cancer (NSCLC) remains a major cause of cancer-related mortality, and safer combination strategies with improved antitumor efficacy are still needed. Berberine (BBR), a natural isoquinoline alkaloid with multi-target pharmacological activity, and celecoxib (CXB), a selective cyclooxygenase-2 (COX-2) inhibitor, have shown antitumor potential. However, their combined efficacy and underlying mechanisms in NSCLC remain unclear.

**Methods:**

Network pharmacology, molecular docking, Chou–Talalay combination index analysis, complementary synergy analysis using SynergyFinder+, *in vitro* functional assays, molecular validation, and a PC9 xenograft model were integrated to evaluate the antitumor effects and associated molecular changes of BBR combined with CXB. Candidate targets and pathways were screened using protein–protein interaction analysis, GO/KEGG enrichment, and Target–Pathway network analysis. Molecular docking was performed to assess the predicted binding affinity of BBR and CXB with key targets, and native-ligand re-docking was performed to validate the docking protocol. Cell viability, proliferation, colony formation, migration, invasion, apoptosis, and molecular changes were examined using CCK-8, EdU, colony formation, wound-healing, Transwell invasion, flow cytometry, Western blotting, qRT-PCR, immunohistochemistry, and *in vivo* experiments.

**Results:**

Network analysis identified EGFR, AKT1, and PTGS2 (encoding COX-2) as candidate nodes, and molecular docking suggested predicted binding of BBR and CXB with EGFR, AKT1, and COX-2. Native-ligand re-docking showed acceptable reproduction of the crystallographic ligand poses. BBR and CXB synergistically reduced A549 and PC9 cell viability, with ZIP, HSA, Loewe, and Bliss analyses further supporting the multidose synergy, and a representative working combination dose was used for validation. The combination significantly inhibited proliferation, colony formation, migration, and invasion while promoting apoptosis. These effects were accompanied by suppression of EGFR and AKT phosphorylation, reduced COX-2 expression, downregulation of TNF-α, IL-1β, and CXCL8, decreased the Bcl-2/Bax ratio, and increased cleaved-Caspase-3. *In vivo*, BBR + CXB more effectively inhibited PC9 xenograft growth than either monotherapy without obvious body weight loss, accompanied by reduced Ki-67, p-EGFR, p-AKT, and COX-2 expression and increased cleaved-Caspase-3 staining.

**Conclusion:**

BBR combined with CXB exhibits synergistic antitumor activity in NSCLC cells and enhanced antitumor efficacy *in vivo*, associated with suppression of EGFR/AKT/COX-2-related signaling, attenuation of inflammatory responses, and activation of apoptosis, providing support for this potential combination strategy.

## 1 Introduction

Lung cancer remains one of the most common malignancies worldwide and ranks among the leading causes of cancer-related death. Non-small cell lung cancer (NSCLC) accounts for the majority of lung cancer cases and continues to represent a major cause of tumor-related mortality (Smolarz et al., 2025; World Health Organization, 2024). Although advances in low-dose computed tomography (CT) screening, molecular targeted therapy, and immunotherapy have substantially improved the diagnosis and treatment of some patients with NSCLC, a considerable proportion of patients are still diagnosed at an intermediate or advanced stage, when opportunities for curative treatment are limited (Oncology Society of Chinese Medical Association, 2024). Moreover, current radiotherapy, chemotherapy, targeted therapy, and immunotherapy still face challenges such as heterogeneous responses, treatment-related toxicity, drug resistance, and disease recurrence (Meyer et al., 2024; Xiang et al., 2024; Memon et al., 2024). Therefore, safer and more effective combination strategies with potential synergistic effects remain an important direction in NSCLC treatment research.

Celecoxib (CXB) is a selective cyclooxygenase-2 (COX-2) inhibitor. In addition to its anti-inflammatory and analgesic effects, its potential value in cancer prevention and treatment has attracted increasing attention. The COX-2/prostaglandin E2 (PGE_2_) axis participates in NSCLC cell proliferation, angiogenesis, invasion, metastasis, and immune escape, serving as an important molecular link between chronic inflammation and tumor progression (Jin et al., 2023; Liu et al., 2024; Finetti et al., 2020). Previous studies have suggested that CXB may have potential value in lung cancer chemoprevention and in suppressing surgery-associated metastatic progression (Mao et al., 2011; Zhang et al., 2014). By inhibiting COX-2/PGE_2_ signaling, CXB may suppress inflammation-driven tumor progression and has shown antiproliferative and pro-apoptotic effects in several tumor models, particularly in smoking-related lung cancer models (Sakurai et al., 2025; Zhang P. et al., 2019). Compared with traditional non-selective COX inhibitors such as indomethacin and diclofenac, CXB has higher selectivity for COX-2, allowing more targeted inhibition of COX-2-derived tumor-promoting prostaglandin production while avoiding some of the pharmacological effects associated with non-selective COX inhibition (Fornai et al., 2014). However, high-dose or long-term CXB administration may still cause dose-related adverse effects, limiting its broader application as an antitumor agent (Flausino et al., 2024). Thus, identifying combination regimens that enhance CXB efficacy while potentially allowing dose optimization is of considerable significance.

Natural bioactive compounds are promising candidates for cancer combination therapy because of their multi-target regulatory properties and broad pharmacological activities. Plant-derived phytochemicals may exert anticancer effects by inhibiting proliferation, inducing apoptosis, modulating oxidative stress, and interfering with tumor-related signaling pathways, and they have shown potential in cancer chemoprevention and adjuvant therapy (Aljabali et al., 2025; Lekhak and Bhattarai, 2024; Qin et al., 2024). Berberine (BBR) is a natural isoquinoline alkaloid derived from traditional Chinese medicinal herbs such as *Coptis chinensis*, with multiple pharmacological activities, including anti-inflammatory, antioxidant, metabolic regulatory, and antitumor effects (Xiong et al., 2022; Rauf et al., 2021). In NSCLC research, BBR has been reported to regulate cell proliferation, apoptosis, autophagy, glycolysis, and metastasis; modulate key oncogenic molecules such as KIF20A and CCNE2; and interfere with tumor-related signaling pathways, including PI3K/AKT and MAPK (Wang Q. et al., 2023; Gu et al., 2022; Hsu et al., 2024). In addition, BBR has been reported to alleviate chemotherapy-related oxidative stress, mitochondrial dysfunction, and inflammatory injury in several toxicity models, suggesting its potential protective effects in specific treatment-related injury models (Wang Y. et al., 2023). CXB mainly acts on the COX-2/PGE_2_-related inflammatory axis, whereas BBR has broader multi-pathway regulatory properties (Kuang et al., 2025; Zhou et al., 2025). Therefore, BBR and CXB may have functional overlap in anti-inflammatory activity, apoptosis induction, and suppression of malignant tumor phenotypes, while also exhibiting potential target-level complementarity.

However, systematic studies on the combined application of BBR and CXB in NSCLC remain limited, and their potential synergistic relationship, key therapeutic targets, and molecular mechanisms have not been fully clarified. Network pharmacology and molecular docking can prioritize potential drug targets and pathways from systems-level network and structural binding perspectives, but these findings still require validation through *in vitro* and *in vivo* experiments (Zhang R. et al., 2019; Boezio et al., 2017). Therefore, this study integrated network pharmacology, molecular docking, Chou-Talalay drug synergy analysis, complementary multidose synergy analysis, *in vitro* functional assays, molecular validation, and a xenograft tumor model to evaluate combined antitumor effects, *in vitro* synergistic interaction, and associated molecular changes of BBR combined with CXB in NSCLC. Specifically, we focused on whether this combination is associated with changes in EGFR/AKT/COX-2-related signaling alongside effects on NSCLC cell proliferation, migration, invasion, and apoptosis. By identifying key targets and pathways, this study aims to provide experimental evidence and molecular insight for the combined application of BBR and CXB in NSCLC and to support the development of network pharmacology-guided combination strategies.

## 2 Methods

### 2.1 Network pharmacology analysis

The overall study workflow is shown in Figure 1. A network pharmacology strategy was used to predict potential targets of BBR and CXB in NSCLC and explore their potential combined regulatory profiles. The names “berberine” and “celecoxib” were used as query terms to retrieve drug-related targets of BBR and CXB from ChEMBL (https://www.ebi.ac.uk/chembl), DrugBank (https://go.drugbank.com/), and TCMSP (https://tcmsp-e.com/tcmsp.php). Because BBR and CXB were prespecified study compounds, oral bioavailability and drug-likeness thresholds were not applied. Only human proteins with explicit compound–target annotations were retained. All candidate targets were standardized using UniProt (https://www.uniprot.org/), with the species restricted to *Homo sapiens*, and converted to official gene symbols. Reviewed human protein entries with valid official gene symbols were retained, and duplicate targets were removed.

**FIGURE 1:**
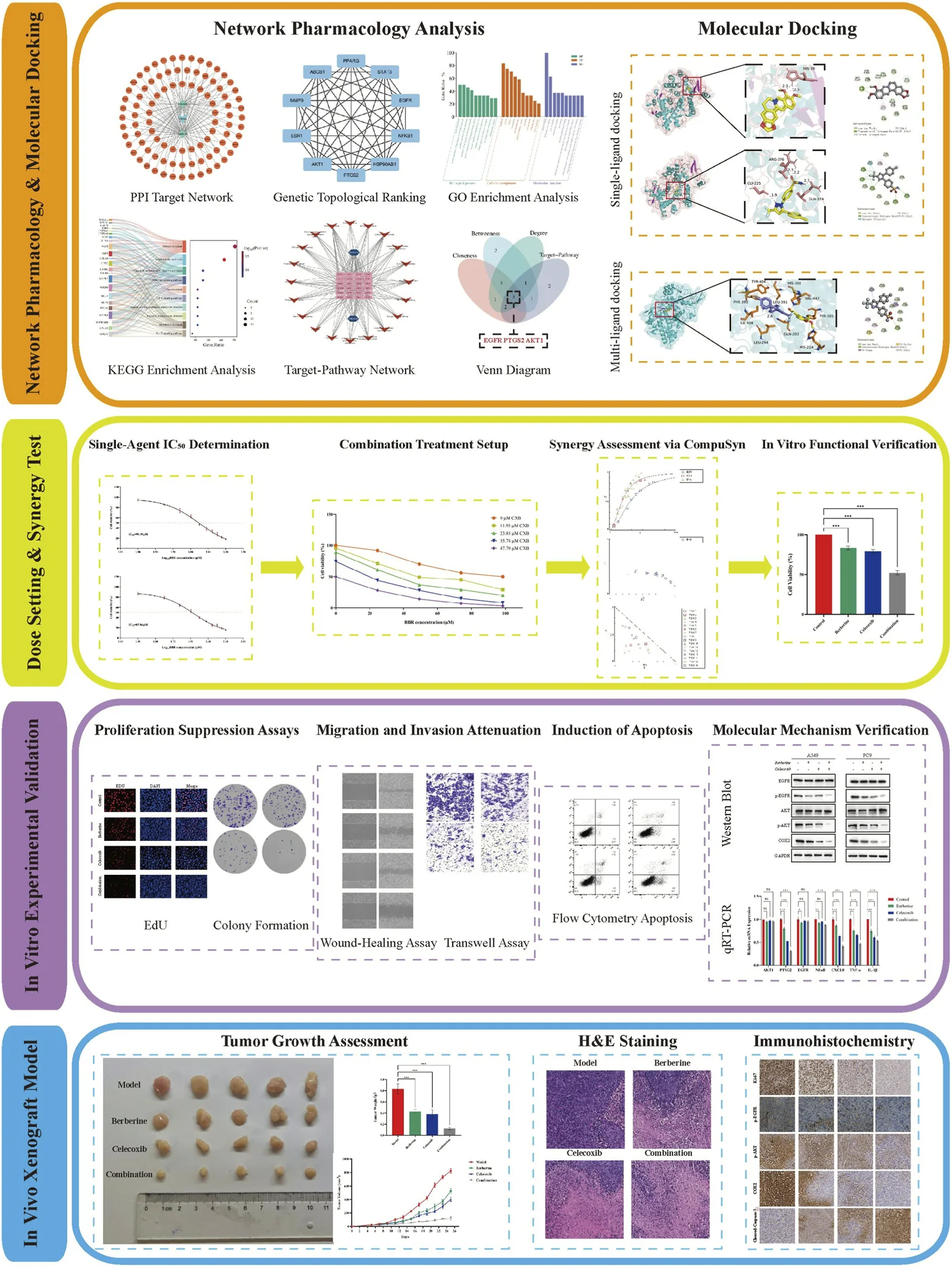
Workflow of the integrated strategy for investigating the combined antitumor effects of BBR and CXB in NSCLC.

NSCLC-associated targets were collected from GeneCards (https://www.genecards.org/) and OMIM (https://www.omim.org/) using “non-small cell lung cancer” as the search term. For GeneCards, targets with relevance scores greater than the median of all retrieved records were retained. For OMIM, which does not provide an equivalent quantitative relevance score, all genes with an explicit association with NSCLC were retained. Intersection analysis was performed among BBR-related targets, CXB-related targets, and NSCLC-associated targets. Targets shared by BBR, CXB, and NSCLC were defined as shared candidate targets. Targets overlapping between BBR and NSCLC but not CXB, and those overlapping between CXB and NSCLC but not BBR, were defined as BBR-related and CXB-related complementary targets, respectively.

A drug-disease-target interaction network was constructed using Cytoscape (version 3.10.1; https://cytoscape.org/). Shared candidate targets were imported into STRING (https://string-db.org/) to construct a PPI network, with the organism set to *Homo sapiens* and the minimum required interaction score set to 0.400. The resulting network was imported into Cytoscape and further analyzed using the CentiScaPe 2.2 plugin. Degree, betweenness centrality, and closeness centrality were calculated and the top 10 targets ranked by each parameter were selected for topological analysis.

GO and KEGG enrichment analyses of the shared candidate targets were performed using DAVID (https://david.ncifcrf.gov/), with *Homo sapiens* selected as the background species. GO terms were categorized into biological process, cellular component, and molecular function. Terms with P < 0.05 were considered significantly enriched. Venn diagrams and enrichment plots were generated using Bioinformatics (https://www.bioinformatics.com.cn/). Based on KEGG results, a Target-Pathway network was constructed in Cytoscape. The top 10 targets ranked by degree in the Target-Pathway network were selected. Core candidate targets were selected by intersecting the top-ranked targets obtained from degree, betweenness centrality, closeness centrality, and Target-Pathway network analyses for subsequent docking and experimental validation.

### 2.2 Molecular docking

Molecular docking was performed to evaluate the potential binding interactions of BBR and CXB with core candidate targets. The three-dimensional structures of receptor proteins were obtained from the Protein Data Bank (PDB; https://www.rcsb.org/), and the structures of BBR and CXB were downloaded from PubChem (https://pubchem.ncbi.nlm.nih.gov/).

Receptor and ligand preprocessing was performed using AutoDockTools (https://autodock.scripps.edu/). Water molecules, original ligands, and unnecessary small molecules were removed. Hydrogen atoms and Gasteiger charges were added, non-polar hydrogens were merged, and receptors and ligands were saved in PDBQT format. For validation of the docking procedure, the native ligand was removed from each crystallographic complex and re-docked into its original binding region using the same docking workflow. The root-mean-square deviation (RMSD) between the re-docked and crystallographic ligand poses was calculated, with an RMSD <2.0 Å considered acceptable for pose reproduction.

Docking was conducted using AutoDock Vina (version 1.2.0; https://vina.scripps.edu/). The grid box was set according to the known active pocket, co-crystallized ligand-binding region, or predicted binding region of each protein. Single-ligand docking was first performed for BBR and CXB separately. A dual-ligand docking system was then constructed by placing BBR and CXB simultaneously within the candidate binding region of the same receptor to evaluate their spatial coexistence within the predicted binding region.

Docking results were assessed according to binding energy, with lower values indicating more favorable predicted binding affinity. Representative docking models with low binding energy and reasonable conformation were selected. Docking conformations were visualized using PyMOL (version 2.3.0; https://pymol.org/), and two-dimensional interactions were analyzed using Discovery Studio 2019 (https://www.3ds.com/products/biovia/discovery-studio). Docking scores and conformations were interpreted as computational predictions rather than evidence of direct target engagement or functional inhibition.

### 2.3 Drugs and reagents

BBR (berberine hemisulfate salt; B3412), CXB (celecoxib; SML3031), and DMSO (D2650) were purchased from Sigma-Aldrich (United States). The purity of BBR and CXB was ≥95% and ≥98%, respectively. BBR and CXB were dissolved in DMSO to prepare 50 mM stock solutions and stored at −20 °C in the dark. Before use, stock solutions were diluted to the required working concentrations.

For all *in vitro* experiments, the final DMSO concentration was maintained below 0.1%, and vehicle controls received an equal volume of DMSO. In combination groups, BBR and CXB were added simultaneously. The representative working combination dose was selected based on cell viability and multidose synergy analyses.

### 2.4 Cell lines and cell culture conditions

A549 and PC9 cells were purchased from the Cell Bank of the Chinese Academy of Sciences (Shanghai, China). Cells were cultured in DMEM supplemented with 10% fetal bovine serum and 1% penicillin/streptomycin at 37 °C in a humidified incubator with 5% CO_2_. All cells were used in the logarithmic growth phase. *Mycoplasma* contamination was excluded before experiments, and passage numbers were controlled within an appropriate range.

### 2.5 Cell viability assay (CCK-8)

Cell viability was assessed using the CCK-8 assay (Beyotime, China). A549 and PC9 cells were seeded into 96-well plates at 1 × 10^4^ cells/well and cultured for 24 h. Cells were then treated with BBR or CXB for 48 h. BBR concentrations were 0–200 μM and CXB concentrations were 0–160 μM, both with 20 μM intervals. The 0 μM group received DMSO as vehicle control, and blank wells were used for background correction.

After treatment, 10 μL CCK-8 solution was added to each well and incubated for 1 h in the dark. Absorbance was measured at 450 nm. Cell viability was calculated as:

$$\text{Cell viability }\left(\%\right)=\left[\left(\text{OD treatment}-\text{OD blank}\right)\;/\;\left(\text{OD vehicle}-\text{OD blank}\right)\right]*100\%$$

Dose-response curves and IC_50_ values were generated using GraphPad Prism. Experiments were performed at least three times with technical replicates.

### 2.6 Drug combination treatment and synergy analysis

Combination experiments were designed based on IC_50_ values obtained from single-agent CCK-8 assays. For each cell line, BBR and CXB were combined at 0%, 25%, 50%, 75%, and 100% of their respective IC_50_ levels in a 5 × 5 dose matrix. The 0% conditions represented the vehicle control or corresponding single-agent treatments, whereas the four non-zero dose levels were fully crossed to generate 16 dual-drug combination conditions. Cells were treated with BBR, CXB, or BBR + CXB for 48 h, and viability was measured as described above.

Drug interactions were evaluated using the Chou–Talalay method, and CI was calculated using CompuSyn software (Chou, 2006). CI < 1, CI = 1, and CI > 1 indicated synergistic, additive, and antagonistic effects, respectively. Dose-effect curves, Fa-CI plots, and isobolograms were generated. The multidose combination matrix was additionally analyzed using SynergyFinder + according to the Bliss, zero interaction potency (ZIP), highest single agent (HSA), and Loewe reference models. The representative working combination dose was selected based on synergistic interaction, sufficient viability inhibition, and avoidance of excessive cell death that could confound subsequent functional assays. Accordingly, 40 μM BBR plus 30 μM CXB was selected for subsequent experiments.

### 2.7 Cell proliferation and colony formation assays

Cell proliferation was evaluated using an EdU detection kit (Beyotime, China). A549 and PC9 cells were seeded into 24-well plates and treated with vehicle, BBR, CXB, or BBR + CXB for 48 h. EdU working solution was added for 2 h, followed by fixation, permeabilization, click reaction, and DAPI counterstaining. Images were captured using a fluorescence microscope, and EdU-positive cells were quantified from randomly selected fields.

For colony formation assays, cells were seeded into 6-well plates at 1,000 cells/well and treated for 48 h. The medium was then replaced with fresh complete medium, and cells were cultured until visible colonies formed. Colonies were fixed with methanol, stained with crystal violet, photographed, and counted. Cell clusters containing more than 50 cells were defined as colonies. Experiments were repeated at least three times.

### 2.8 Migration and invasion assays

For wound-healing assays, A549 and PC9 cells were cultured in 6-well plates until confluent. A straight scratch was made with a sterile 200 μL pipette tip, and detached cells were removed. Cells were then cultured in serum-free medium containing vehicle, BBR, CXB, or BBR + CXB. Images were captured at 0 h and 48 h, and wound area was quantified using ImageJ.

For Transwell invasion assays, upper chambers were precoated with diluted Matrigel. After treatment, cells were resuspended in serum-free medium and seeded into the upper chambers, while complete medium containing 10% FBS was added to the lower chambers. After 37–48 h, invaded cells on the lower membrane surface were fixed, stained with crystal violet, photographed, and counted in randomly selected fields.

### 2.9 Apoptosis analysis

Apoptosis was detected using Annexin V-FITC/PI staining followed by flow cytometry. A549 and PC9 cells were treated with vehicle, BBR, CXB, or BBR + CXB for 48 h. Both adherent and floating cells were collected, washed, resuspended in 1× binding buffer, and stained with Annexin V-FITC and PI according to the manufacturer’s instructions. After incubation in the dark for 15 min, samples were analyzed by flow cytometry. Total apoptosis was calculated as the sum of early and late apoptotic cells.

### 2.10 Western blot analysis

A549 and PC9 cells were treated with vehicle, BBR, CXB, or BBR + CXB for 48 h. Total protein was extracted using RIPA lysis buffer containing protease and phosphatase inhibitors, and protein concentration was determined using a BCA kit. Equal amounts of protein were separated by SDS-PAGE and transferred onto PVDF membranes. After blocking with 5% non-fat milk or BSA, membranes were incubated overnight at 4 °C with primary antibodies listed in Supplementary Table S1.

Membranes were incubated with HRP-conjugated goat anti-rabbit IgG H&L secondary antibody and visualized using enhanced chemiluminescence. Images were acquired using a Tanon 5200 system, and band intensities were quantified using ImageJ. Phosphorylated proteins were normalized to their corresponding total proteins, and other target proteins were normalized to GAPDH.

### 2.11 qRT-PCR analysis

Total RNA was extracted after 48 h treatment using TRIzol reagent, and RNA of adequate quality was reverse-transcribed into cDNA. qRT-PCR was performed using an ABI 7500 system with *GAPDH* as the internal reference. The detected genes included *EGFR, AKT1, PTGS2, RELA, CXCL8, TNF*, and *IL1B*. Primer sequences are listed in Supplementary Table S2.

The PCR program was: 95 °C for 3 min, followed by 40 cycles of 95 °C for 30 s, 55 °C for 20 s, and 72 °C for 20 s. Melting curve analysis was performed to confirm specificity. Relative gene expression was calculated using the 2^−ΔΔCt method.

### 2.12 Xenograft tumor model

Six-week-old male BALB/c nude mice were purchased from the Model Animal Research Center of Nanjing University and housed under specific pathogen-free conditions. After 1 week of acclimation, 5 × 10^6^ PC9 cells in 100 μL PBS were subcutaneously injected into the right dorsal flank. When tumors became palpable, mice were randomly divided into four groups with five mice per group: vehicle, BBR, CXB, and BBR + CXB.

BBR and CXB were dissolved in DMSO and diluted with sterile normal saline, with the final vehicle consisting of 10% DMSO in sterile normal saline. Mice received vehicle, BBR 10 mg/kg, CXB 10 mg/kg, or BBR 10 mg/kg plus CXB 10 mg/kg by intraperitoneal injection every other day. Tumor size and body weight were measured every 2 days. Tumor volume was calculated as:

$\text{Tumor volume}=0.5\times D\times d^{2}$ , where D is the long diameter and d is the short diameter.

At the endpoint, mice were euthanized under anesthesia, and tumors were excised, weighed, and fixed in 10% neutral formalin. Tumor measurements and tissue processing were performed by blinded investigators. Animal experiments were approved by the Institutional Animal Care and Use Committee of Nanjing Medical University.

### 2.13 Histology and immunohistochemistry

Tumor tissues were fixed in 10% neutral buffered formalin, embedded in paraffin, and sectioned at approximately 4 μm. H&E staining was performed to evaluate histological changes. For immunohistochemistry, sections were deparaffinized, rehydrated, subjected to antigen retrieval in citrate buffer, blocked with 3% hydrogen peroxide and normal goat serum, and incubated overnight at 4 °C with primary antibodies listed in Supplementary Table S1. HRP-conjugated goat anti-rabbit IgG H&L was used as the secondary antibody. Immunoreactivity was visualized using DAB and counterstained with hematoxylin.

For each specimen, five randomly selected high-power fields were analyzed. Staining was quantified using ImageJ and expressed as the percentage of positive cells, positive staining area, or mean optical density as appropriate for the staining characteristics. Quantification was performed by blinded investigators.

### 2.14 Statistical analysis

All quantitative data are expressed as the mean ± SD from at least three independent experiments. Statistical analyses were performed using GraphPad Prism 8.0. Comparisons between two groups were conducted using Student’s t-test, whereas multiple-group comparisons were performed using one-way ANOVA followed by Tukey’s test when appropriate. Time-dependent data, including tumor volume and body weight, were analyzed using two-way ANOVA.

Drug interactions were evaluated using the Chou-Talalay method, and CI was calculated using CompuSyn. Complementary multidose synergy analyses were performed using SynergyFinder + based on the Bliss, ZIP, HSA, and Loewe reference models. All tests were two-sided, and P < 0.05 was considered statistically significant. Figure symbols were defined as follows: *P < 0.05, **P < 0.01, and ***P < 0.001.

## 3 Result

### 3.1 Network pharmacology analysis identifies shared and complementary candidate targets of BBR and CXB in NSCLC

The chemical structures of BBR and CXB are shown in Figures 2A,B. After integrating drug-related targets and NSCLC-associated disease targets from multiple databases, Venn analysis was performed to identify the overlapping target sets (Figure 2C). A total of 47 targets were shared among BBR, CXB, and NSCLC and were defined as shared candidate targets. In addition, 29 targets overlapped between BBR and NSCLC but not CXB, whereas 17 targets overlapped between CXB and NSCLC but not BBR, representing BBR-related and CXB-related complementary targets, respectively. The full target lists are provided in Supplementary Table S3. These results suggest that BBR and CXB may be associated with both shared molecular targets and drug-specific complementary target profiles in NSCLC.

**FIGURE 2:**
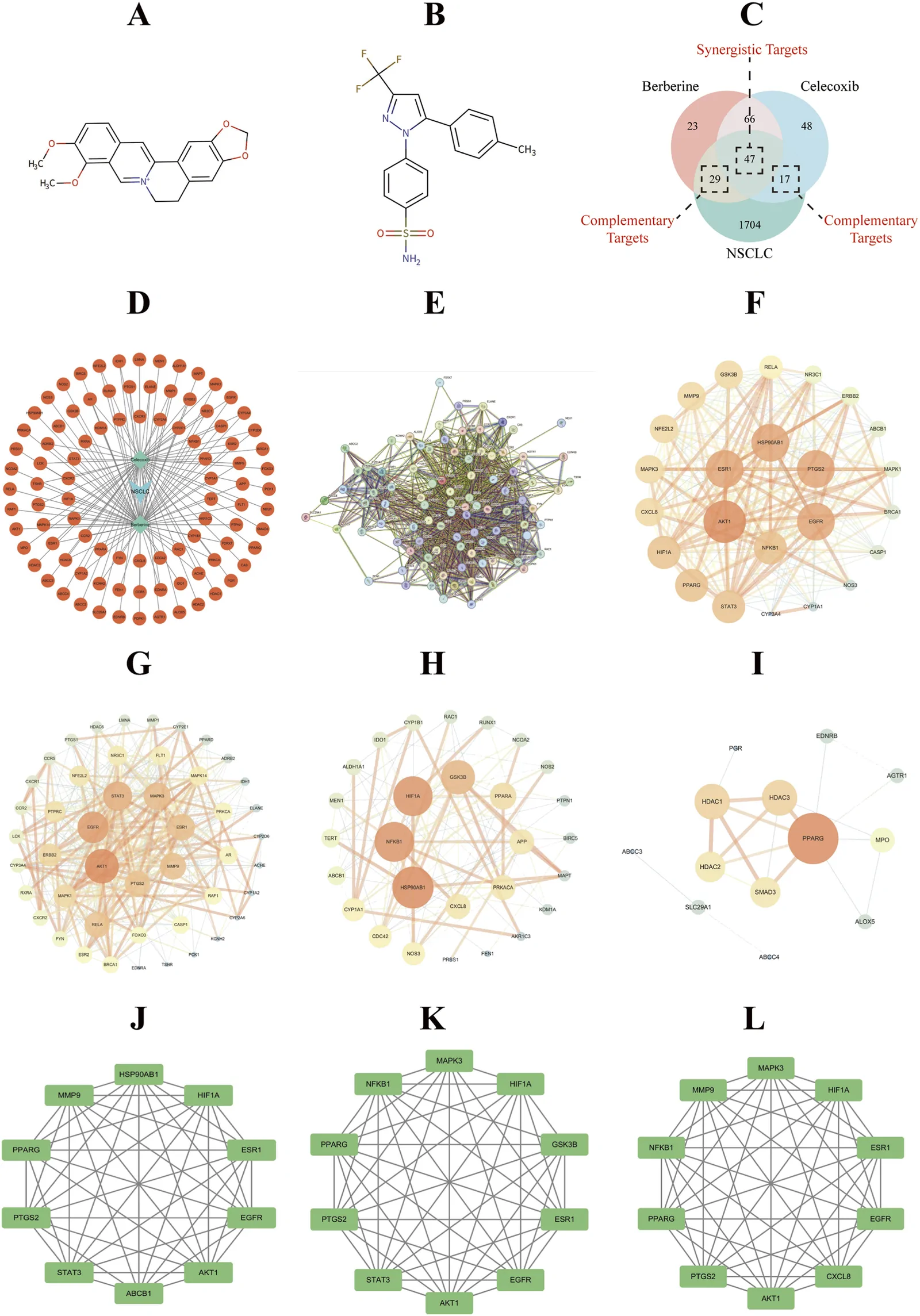
Network pharmacology analysis identifies shared and complementary targets of BBR and CXB in NSCLC. **(A,B)** Chemical structures of berberine (BBR) and celecoxib (CXB). **(C)** Venn diagram showing the intersection among BBR-related targets, CXB-related targets, and NSCLC-associated targets. **(D)** Drug–disease–target interaction network of BBR, CXB, NSCLC, and candidate targets. **(E)** Global protein–protein interaction (PPI) network constructed from the shared candidate targets. **(F)** Hub-target subnetwork extracted from the global PPI network based on topological ranking. **(G–I)** PPI subnetworks of shared candidate targets, BBR-related complementary targets, and CXB-related complementary targets. **(J–L)** Top-ranked hub targets identified by betweenness centrality, closeness centrality, and degree. BBR, berberine; CXB, celecoxib; NSCLC, non-small cell lung cancer; PPI, protein–protein interaction.

A drug-disease-target interaction network was then constructed to visualize the associations among BBR, CXB, NSCLC, and candidate targets (Figure 2D). Several targets were simultaneously connected with both drugs and NSCLC, illustrating shared predicted drug–disease target associations. The 47 shared candidate targets were further imported into STRING to construct a PPI network (Figure 2E). This network showed relatively dense protein interactions, suggesting potential functional connectivity among these candidate targets. A hub-target subnetwork was then extracted according to topological characteristics (Figure 2F).

To compare the network features of different target subsets, PPI subnetworks were separately constructed for the shared targets, BBR-related complementary targets, and CXB-related complementary targets (Figures 2G–I). The shared target subnetwork showed a more concentrated interaction pattern, whereas the BBR- and CXB-related complementary subnetworks reflected drug-specific target extensions. These findings suggest that the predicted target profiles of BBR and CXB comprise both shared and complementary components.

Topological ranking was further performed using betweenness centrality, closeness centrality, and degree (Figures 2J–L). In the top 10 lists, AKT1 ranked first in closeness, betweenness, and degree, while EGFR and PTGS2 also appeared among the highly ranked targets across multiple metrics. Specifically, EGFR ranked fourth in closeness, seventh in betweenness, and fourth in degree, whereas PTGS2 ranked 10th in closeness, eighth in betweenness, and second in degree (Supplementary Table S4). Together, these results identified EGFR, AKT1, and PTGS2 as candidate hub targets for subsequent enrichment, docking, and experimental validation.

### 3.2 GO/KEGG enrichment and Target-Pathway network analyses highlight EGFR/AKT/PTGS2-related signaling

GO and KEGG enrichment analyses were performed to clarify the biological functions and pathways associated with the shared candidate targets. GO analysis showed that these targets were mainly enriched in biological processes related to tumor progression and inflammatory regulation, including response to oxidative stress, cellular response to chemical stimulus, inflammatory response, regulation of apoptosis, epithelial cell proliferation, and response to lipopolysaccharide (Figure 3A). Cellular component terms were mainly associated with the plasma membrane, membrane raft/microdomain, cytoplasm, and extracellular region, while molecular function terms included receptor ligand activity, cytokine receptor binding, enzyme binding, and protein kinase-related regulation.

**FIGURE 3:**
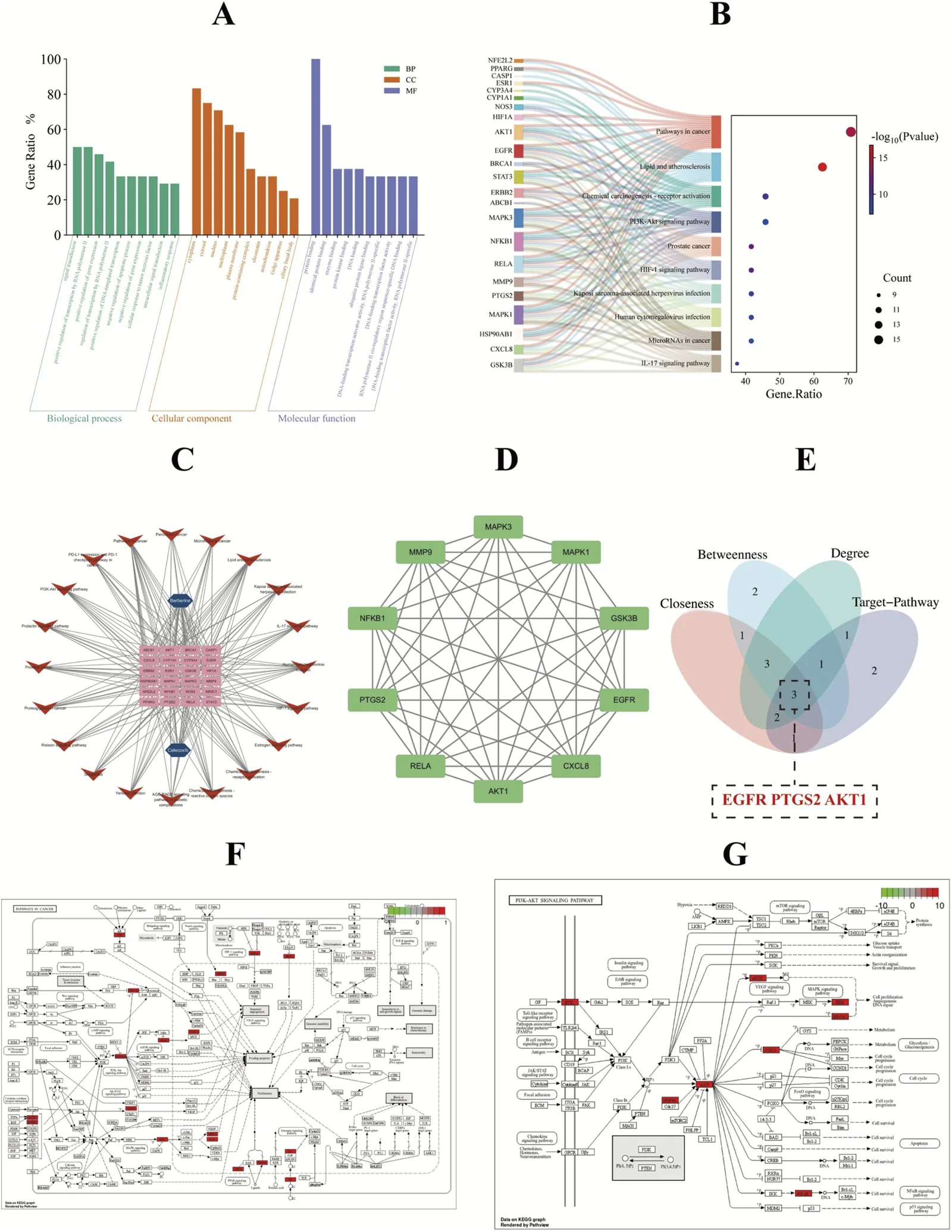
Enrichment and Target–Pathway analyses identify EGFR/AKT/PTGS2-related signaling. **(A)** GO enrichment analysis of shared candidate targets, including biological process, cellular component, and molecular function terms. **(B)** KEGG pathway enrichment analysis of shared candidate targets. **(C)** Target–Pathway interaction network showing the relationships between candidate targets and enriched pathways. **(D)** Top-ranked targets identified based on Target–Pathway network topology. **(E)** Venn diagram showing the intersection of four screening strategies: betweenness centrality, closeness centrality, degree, and Target–Pathway ranking. **(F,G)** KEGG pathway maps showing the distribution of candidate targets in pathways in cancer and the PI3K-Akt signaling pathway. GO, Gene Ontology; KEGG, Kyoto Encyclopedia of Genes and Genomes; NSCLC, non-small cell lung cancer; PTGS2, prostaglandin-endoperoxide synthase 2.

KEGG enrichment analysis further showed that the shared targets were enriched in multiple cancer- and inflammation-associated pathways, including pathways in cancer, PI3K-Akt signaling pathway, HIF-1 signaling pathway, IL-17 signaling pathway, TNF signaling pathway, NF-κB signaling pathway, and JAK-STAT signaling pathway (Figure 3B). These pathways are closely associated with tumor cell proliferation, survival, inflammatory responses, invasion, and therapeutic resistance, suggesting that BBR and CXB may be associated with multiple interconnected oncogenic and inflammatory pathways in NSCLC.

A Target-Pathway interaction network was then constructed to integrate enriched pathways and their corresponding targets (Figure 3C). Target-Pathway ranking identified several targets that participated in multiple enriched pathways (Figure 3D). The top-ranked Target-Pathway nodes included MAPK1, MAPK3, NFKB1, AKT1, RELA, EGFR, PTGS2, GSK3B, CXCL8, and MMP9 (Supplementary Table S4), suggesting that these targets may occupy important positions within the predicted pathway network.

To further refine candidate targets, Venn intersection analysis was performed using four screening strategies: betweenness centrality, closeness centrality, degree, and Target-Pathway ranking (Figure 3E). EGFR, AKT1, and PTGS2 were located at the intersection of these four ranking approaches, indicating that they had high centrality not only in the PPI network but also in the pathway network. Further KEGG pathway mapping showed that these targets were distributed within pathways in cancer and the PI3K-Akt signaling pathway (Figures 3F,G). In particular, EGFR and AKT1 were mapped to receptor-linked intracellular kinase signaling, whereas PTGS2 (encoding COX-2) represented an inflammation-related candidate node. These findings prioritized EGFR/AKT/PTGS2-related signaling as a candidate molecular framework for further validation.

### 3.3 Molecular docking suggests potential interactions with EGFR, AKT1, and COX-2

Molecular docking was performed to evaluate the potential binding patterns of BBR and CXB with EGFR, AKT1, and COX-2. Docking results were assessed using binding energy scores generated by AutoDock Vina, with lower binding energy indicating more favorable predicted binding affinity. Based on commonly used empirical criteria in molecular docking studies, a binding energy below −5.0 kcal/mol was considered to indicate favorable predicted binding affinity (Wang et al., 2022; Wang C. et al., 2025).

To assess the ability of the docking procedure to reproduce experimentally observed ligand poses, native-ligand re-docking was additionally performed for EGFR, AKT1, and COX-2. The re-docked poses showed close overlap with the corresponding crystallographic ligand conformations (Supplementary Figure S1), with RMSD values of 0.319, 0.581, and 0.647 Å, respectively (Supplementary Table S5). All RMSD values were below the predefined 2.0 Å criterion, supporting acceptable reproduction of the crystallographic ligand poses.

Single-ligand docking showed that BBR could be accommodated within the candidate binding pockets of EGFR, AKT1, and COX-2 and form hydrogen bonds, hydrophobic interactions, and other non-covalent interactions with key residues (Figures 4A–C). The binding energies of BBR with EGFR, AKT1, and COX-2 were −7.60, −10.40, and −7.80 kcal/mol, respectively. CXB also showed favorable predicted binding with the same targets (Figures 4D–F), with binding energies of −6.90, −10.30, and −7.80 kcal/mol for EGFR, AKT1, and COX-2, respectively. These values were all below −5.0 kcal/mol, suggesting favorable predicted binding affinities.

**FIGURE 4:**
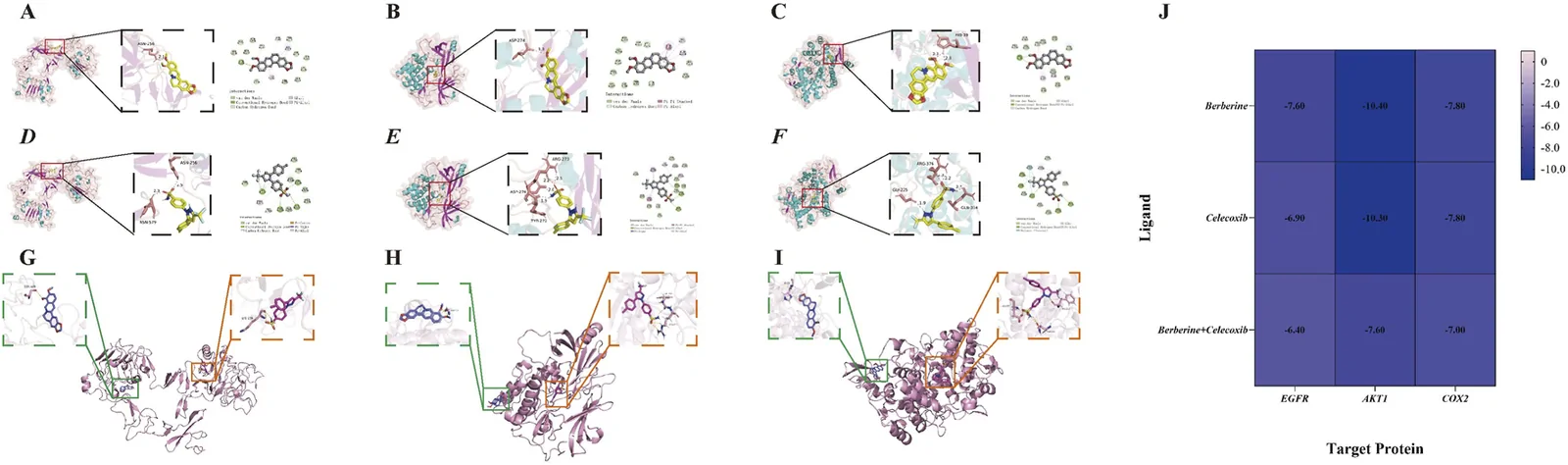
Molecular docking analysis of BBR and CXB with EGFR, AKT1, and COX-2. **(A–C)** Predicted docking conformations of BBR with EGFR, AKT1, and COX-2. **(D–F)** Predicted docking conformations of CXB with EGFR, AKT1, and COX-2. **(G–I)** Dual-ligand docking models showing the potential spatial coexistence of BBR and CXB within the candidate binding regions of EGFR, AKT1, and COX-2. **(J)** Heatmap summarizing the predicted binding energies of BBR, CXB, and BBR + CXB with EGFR, AKT1, and COX-2. Lower binding energy indicates stronger predicted binding affinity and a more stable docking conformation. BBR, berberine; CXB, celecoxib; COX-2, cyclooxygenase-2.

Dual-ligand docking was then performed to explore whether BBR and CXB could coexist within the candidate binding regions of each target protein. The results showed that the two ligands displayed spatial coexistence or adjacent binding patterns within EGFR, AKT1, and COX-2 binding regions (Figures 4G–I). The binding energies of the BBR + CXB dual-ligand systems with EGFR, AKT1, and COX-2 were −6.40, −7.60, and −7.00 kcal/mol, respectively (Figure 4J), indicating that the combined docking systems retained favorable predicted binding affinities under the computational conditions. Among the targets, AKT1-related docking systems generally showed lower binding energies. These docking results further supported the prioritization of EGFR, AKT1, and COX-2 for subsequent experimental validation.

### 3.4 BBR and CXB synergistically reduce NSCLC cell viability

CCK-8 assays were performed to evaluate the effects of BBR and CXB on A549 and PC9 cell viability. Both drugs reduced cell viability in a concentration-dependent manner after 48 h treatment (Figure 5A), and IC_50_ values were calculated from the fitted dose-response curves. Based on these IC_50_ values, different BBR and CXB combination concentrations were designed and tested. The inhibitory effect of the combination treatment increased with increasing drug concentrations in both A549 and PC9 cells (Figure 5B).

**FIGURE 5:**
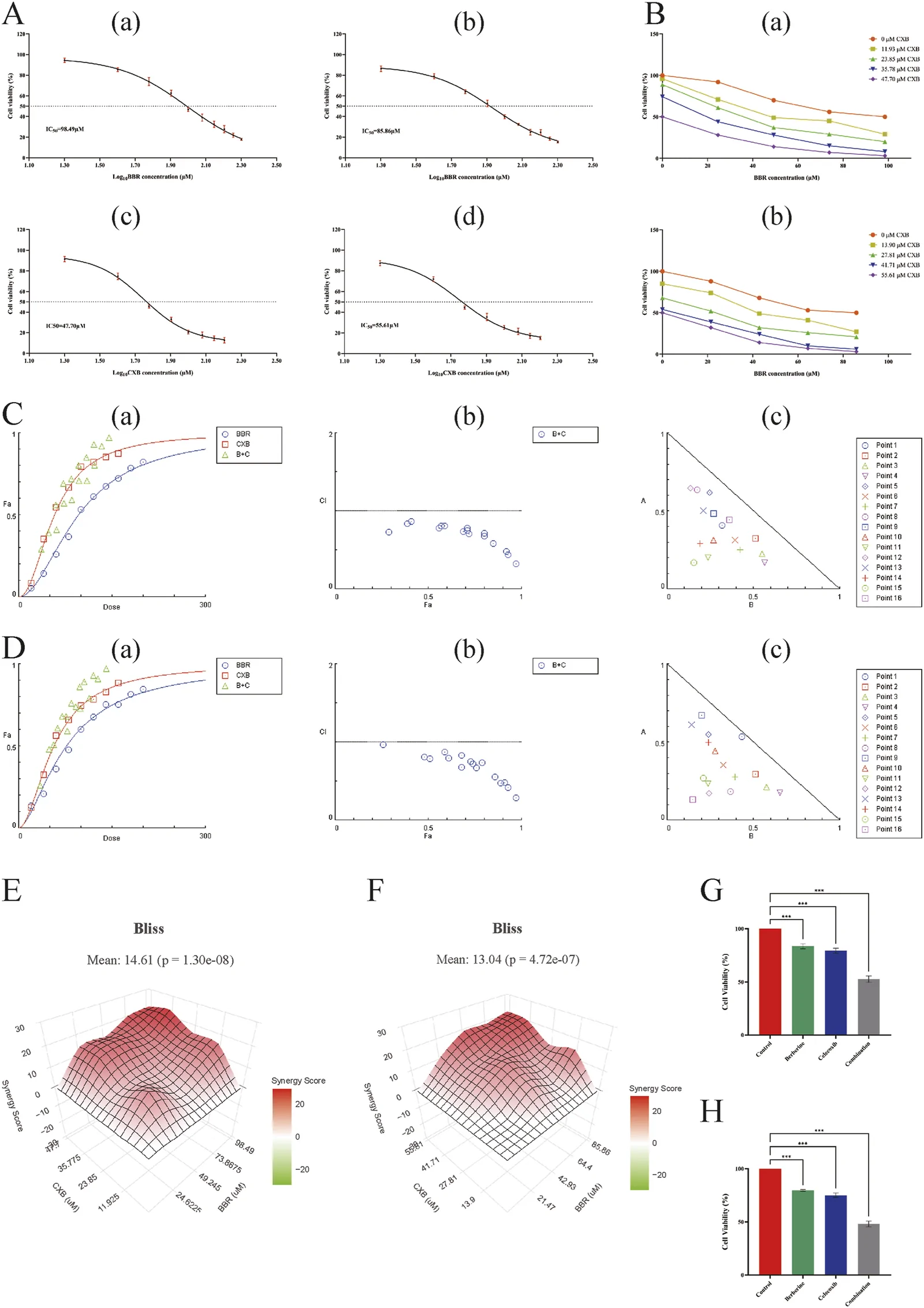
BBR and CXB synergistically reduce NSCLC cell viability and define the representative combination dose. **(A)** Single-agent dose–response curves after 48 h of treatment: (a) BBR in A549 cells, (b) BBR in PC9 cells, (c) CXB in A549 cells, and (d) CXB in PC9 cells. The corresponding IC50 values were determined from the fitted dose–response curves. **(B)** Cell viability under multidose BBR–CXB combination treatment: (a) A549 cells and (b) PC9 cells. **(C)** Chou–Talalay combination analysis in A549 cells: (a) dose–effect curves for BBR, CXB, and BBR+CXB, (b) Fa–CI plot, and (c) isobologram analysis of the 16 tested combination conditions. **(D)** Chou–Talalay combination analysis in PC9 cells: (a) dose–effect curves for BBR, CXB, and BBR+CXB, (b) Fa–CI plot, and (c) isobologram analysis of the 16 tested combination conditions. **(E,F)** Bliss synergy landscapes of the multidose BBR–CXB combination matrices analyzed using SynergyFinder+ in **(E)** A549 and **(F)** PC9 cells. **(G,H)** Cell viability after treatment with vehicle, BBR, CXB, or BBR + CXB at the representative working combination dose in **(G)** A549 and **(H)** PC9 cells. Data are expressed as the mean ± SD from at least three independent experiments. For Chou–Talalay analysis, CI < 1 indicates synergism, CI = 1 indicates an additive effect, and CI > 1 indicates antagonism. Positive Bliss synergy scores indicate that the observed combination effect exceeds the expected effect under the Bliss independence model. *P < 0.05, **P < 0.01, ***P < 0.001. BBR, berberine; CXB, celecoxib; NSCLC, non-small cell lung cancer; CI, combination index; Fa, fraction affected; IC50, half-maximal inhibitory concentration.

To determine whether the combined effect exceeded additivity, Chou-Talalay analysis was performed. Most combination points showed CI values below 1, and the Fa-CI curves were mainly located within the synergistic range. In addition, several combination points in the isobologram were distributed below the line of additivity (Figures 5C,D), indicating synergistic inhibition of cell viability rather than simple additive suppression. To further assess the robustness of the observed interaction, the multidose combination matrices were additionally analyzed using SynergyFinder + based on the Bliss, ZIP, HSA, and Loewe reference models. Bliss analysis yielded positive overall synergy scores in both A549 (14.61) and PC9 (13.04) cells (Figures 5E,F). Consistently, ZIP, HSA, and Loewe analyses also produced positive overall synergy scores in A549 cells (14.53, 27.31, and 17.37, respectively) and PC9 cells (13.08, 28.06, and 17.28, respectively) (Supplementary Figure S2; Supplementary Table S6). These complementary models consistently supported a synergistic interaction across the multidose combination matrices.

For subsequent functional and molecular experiments, the representative working combination dose was selected by considering three factors: clear inhibitory effect, synergistic interaction based on multidose synergy analyses, and avoidance of excessive cell death that could interfere with downstream assays. Based on these criteria, 40 μM BBR plus 30 μM CXB was selected. At this dose, BBR and CXB alone reduced A549 and PC9 cell viability, while BBR + CXB further and significantly decreased cell viability compared with either monotherapy (Figures 5G,H). These results further supported that BBR and CXB synergistically suppressed NSCLC cell viability and provided a rational dose basis for subsequent experiments.

### 3.5 BBR + CXB suppresses malignant phenotypes and promotes apoptosis

Using the representative working combination dose, we next examined the effects of BBR + CXB on NSCLC cell proliferation, clonogenicity, migration, invasion, and apoptosis. EdU assays showed that the proportion of EdU-positive cells was high in the control group, reflecting active DNA synthesis. BBR or CXB alone reduced EdU positivity, whereas BBR + CXB produced the most pronounced reduction in both A549 and PC9 cells (Figures 6A–C), indicating stronger inhibition of proliferative activity.

**FIGURE 6:**
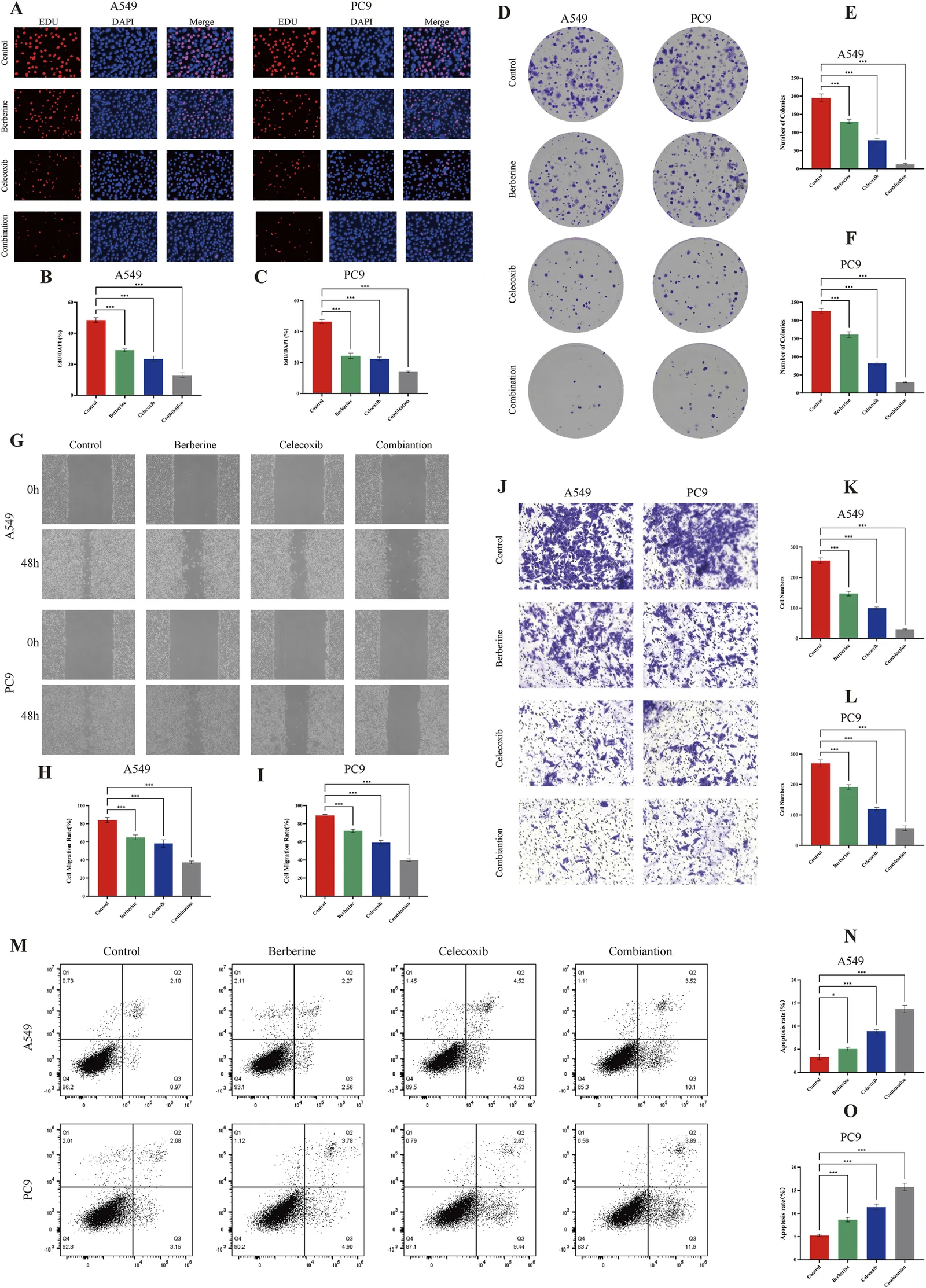
BBR + CXB suppresses malignant phenotypes and promotes apoptosis in NSCLC cells. **(A–C)** EdU assay showing the proliferative activity of A549 and PC9 cells treated with vehicle, BBR, CXB, or BBR + CXB. **(D–F)** Colony formation assay showing the clonogenic ability of A549 and PC9 cells after different treatments. **(G–I)** Wound-healing assay assessing the migratory capacity of A549 and PC9 cells at 0 h and 48 h after treatment. **(J–L)** Transwell invasion assay showing the invasive ability of A549 and PC9 cells after different treatments. **(M–O)** Flow cytometry analysis of apoptosis in A549 and PC9 cells using Annexin V-FITC/PI staining. Data are expressed as the mean ± SD from at least three independent experiments. *P < 0.05, **P < 0.01, ***P < 0.001. BBR, berberine; CXB, celecoxib; NSCLC, non-small cell lung cancer.

Colony formation assays further showed that control cells formed numerous visible colonies, whereas monotherapy reduced colony number to varying degrees. The combination group showed the lowest colony-forming ability (Figures 6D–F), suggesting that BBR + CXB not only suppressed short-term proliferation but also weakened sustained clonogenic potential.

Wound-healing assays showed that control cells displayed obvious wound closure within 48 h. BBR and CXB monotherapy delayed wound closure, while the combination group retained the largest residual wound area (Figures 6G–I). Consistently, Transwell assays showed that the number of invaded cells decreased after monotherapy and was further reduced after BBR + CXB treatment (Figures 6J–L). These results indicated that BBR + CXB more effectively inhibited NSCLC cell migration-related capacity and invasion.

Flow cytometry using Annexin V-FITC/PI staining was performed to assess apoptosis. BBR and CXB monotherapy increased apoptotic rates compared with the control group, whereas BBR + CXB induced a more pronounced shift toward apoptotic quadrants and significantly increased total apoptosis in both cell lines (Figures 6M–O). Overall, BBR + CXB suppressed multiple malignant phenotypes and promoted apoptosis in NSCLC cells.

### 3.6 BBR + CXB is associated with suppression of EGFR/AKT/COX-2 signaling

To explore the molecular changes associated with the functional effects of BBR + CXB, EGFR/AKT/COX-2-related signaling and inflammation- and apoptosis-related molecules were examined. Western blot analysis showed that BBR or CXB alone reduced EGFR and AKT phosphorylation to some extent, while BBR + CXB caused a more obvious decrease in the p-EGFR/EGFR and p-AKT/AKT ratios in A549 and PC9 cells (Figures 7A–C). COX-2 protein expression was also markedly reduced in the combination group (Figures 7A,D). Total EGFR and AKT levels changed only slightly, suggesting that the combination predominantly affected EGFR and AKT phosphorylation rather than total protein abundance.

**FIGURE 7:**
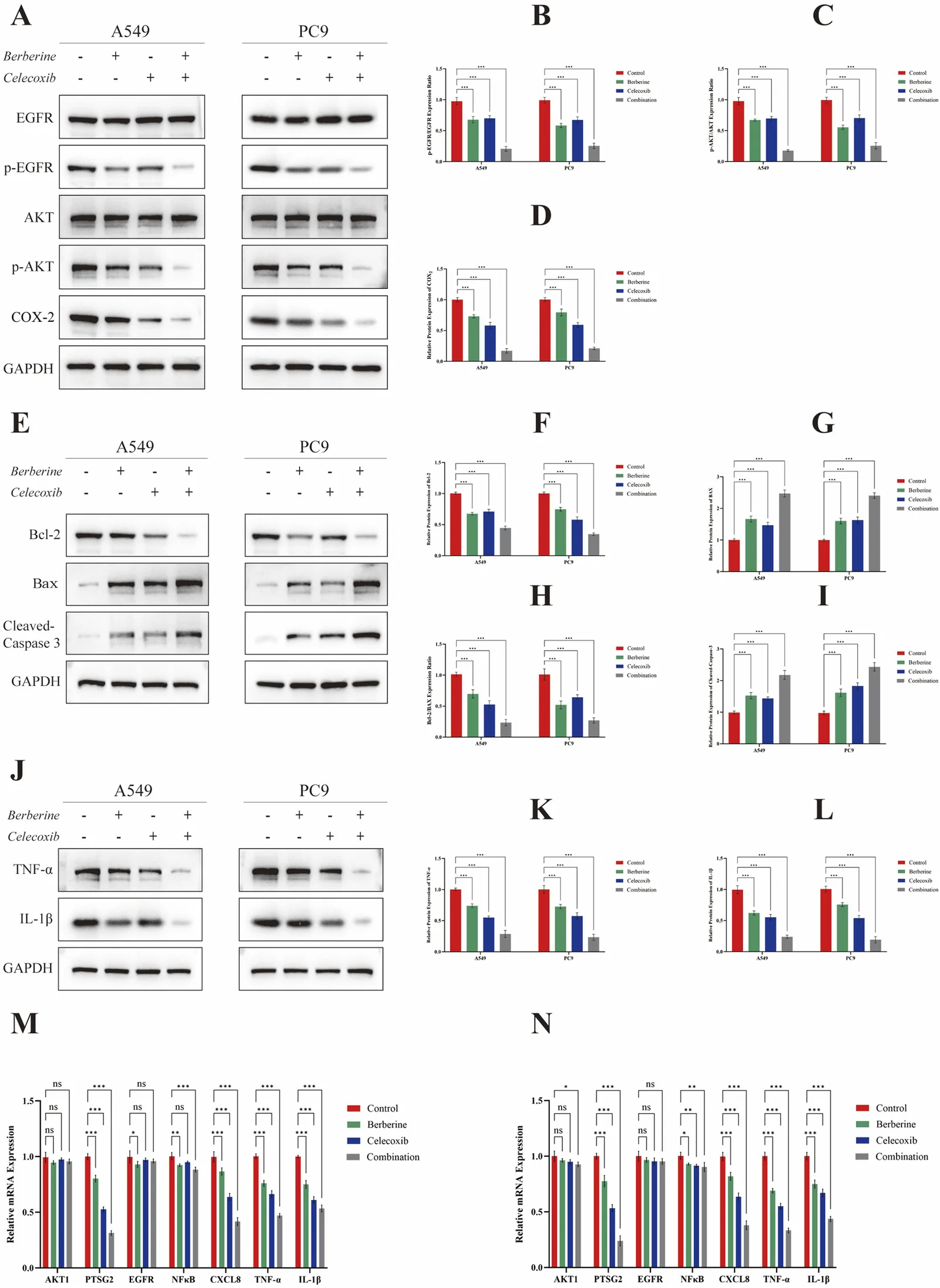
BBR + CXB suppresses EGFR/AKT/COX-2 signaling and modulates inflammation- and apoptosis-related molecules in NSCLC cells. **(A)** Western blot analysis of EGFR, p-EGFR, AKT, p-AKT, COX-2, and GAPDH in A549 and PC9 cells after different treatments. **(B–D)** Quantitative analysis of p-EGFR/EGFR, p-AKT/AKT, and COX-2/GAPDH expression. **(E)** Western blot analysis of Bcl-2, Bax, cleaved-Caspase-3, and GAPDH in A549 and PC9 cells after different treatments. **(F–I)** Quantitative analysis of Bcl-2/GAPDH, Bax/GAPDH, Bcl-2/Bax ratio, and cleaved-Caspase-3/GAPDH expression. **(J)** Western blot analysis of TNF-α, IL-1β, and GAPDH in A549 and PC9 cells after different treatments. **(K,L)** Quantitative analysis of TNF-α/GAPDH and IL-1β/GAPDH expression. **(M,N)** qRT-PCR analysis of AKT1, PTGS2, EGFR, RELA/NF-κB, CXCL8, TNF, and IL1B mRNA expression in A549 and PC9 cells. Data are expressed as the mean ± SD from at least three independent experiments. ns, not significant; *P < 0.05, **P < 0.01, ***P < 0.001. BBR, berberine; CXB, celecoxib; NSCLC, non-small cell lung cancer; COX-2, cyclooxygenase-2.

Apoptosis-related proteins were then analyzed. BBR and CXB alone decreased Bcl-2 and increased Bax to varying degrees, whereas BBR + CXB further reduced Bcl-2, increased Bax, and decreased the Bcl-2/Bax ratio (Figures 7E–H). Cleaved-Caspase-3 expression was also highest in the combination group (Figures 7E,I), consistent with the flow cytometry results.

Inflammation-related proteins were further examined. TNF-α and IL-1β levels decreased after monotherapy and were further reduced after BBR + CXB treatment (Figures 7J–L). qRT-PCR analysis showed that BBR + CXB markedly downregulated *PTGS2, RELA/NF-κB, CXCL8, TNF*, and *IL1B* mRNA levels in both cell lines (Figures 7M,N). In contrast, *EGFR* and *AKT1* mRNA levels changed only modestly, consistent with more pronounced changes in EGFR and AKT phosphorylation than in their mRNA expression. These results indicate that the effects of BBR + CXB were accompanied by suppression of EGFR/AKT/COX-2-related signaling, reduced inflammatory mediator expression, and changes in apoptosis-related molecules.

### 3.7 BBR + CXB inhibits PC9 xenograft tumor growth

To further evaluate the antitumor effect of BBR + CXB *in vivo*, a PC9 xenograft model was established. At the endpoint, tumors from the BBR and CXB monotherapy groups were smaller than those from the vehicle group, while the BBR + CXB group showed the smallest tumors (Figure 8A). Tumor growth curves showed that vehicle-treated tumors grew continuously, whereas monotherapy slowed tumor growth and combination treatment produced the strongest growth inhibition (Figure 8B). Final tumor weight analysis was consistent with tumor volume changes, with the lowest tumor weight observed in the combination group (Figure 8C). No obvious body weight loss was observed among the groups (Figure 8D).

**FIGURE 8:**
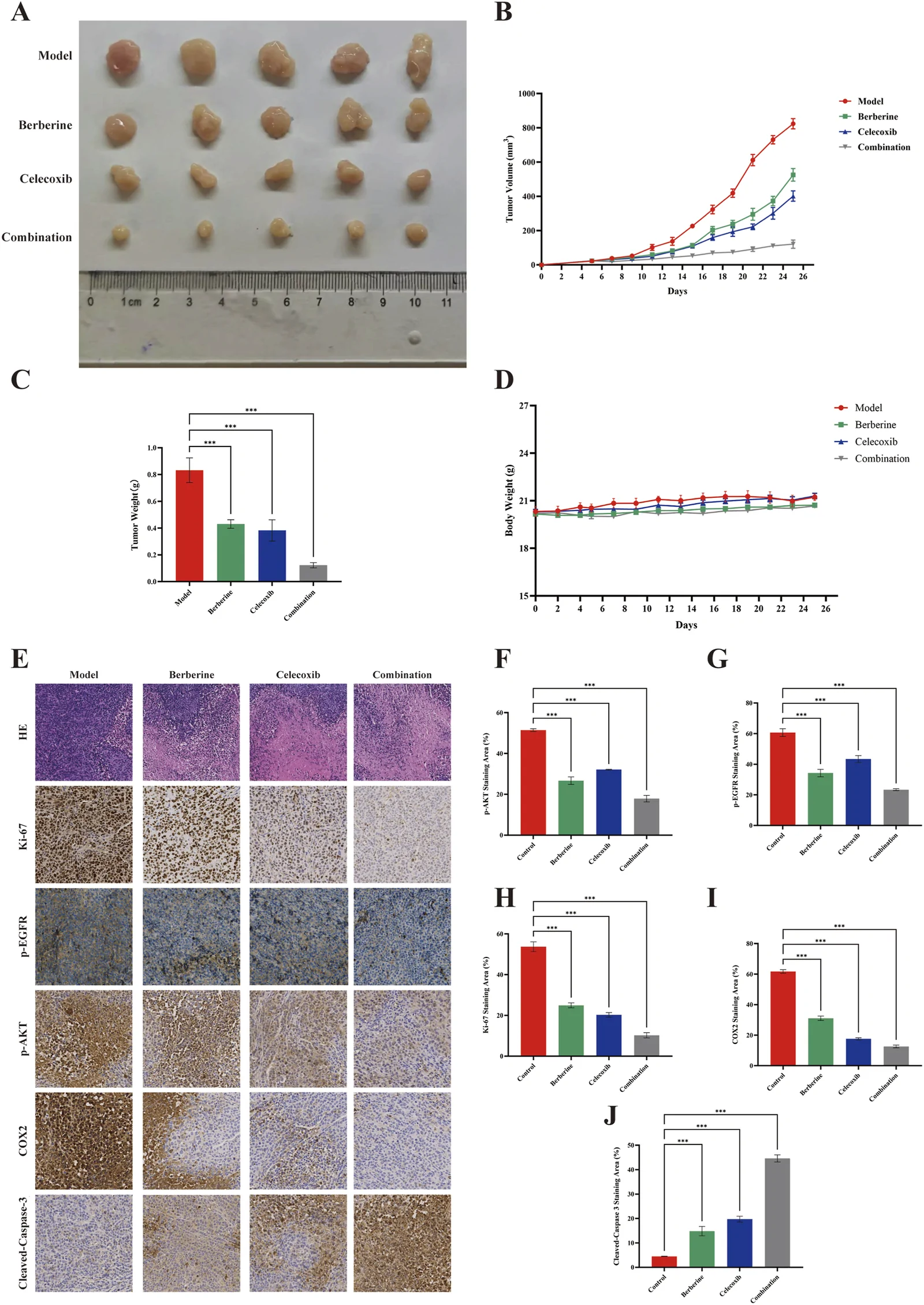
BBR + CXB suppresses PC9 xenograft tumor growth and modulates EGFR/AKT/COX-2 signaling *in vivo*. **(A)** Representative images of excised xenograft tumors from mice treated with vehicle, BBR, CXB, or BBR + CXB. **(B)** Tumor growth curves during the treatment period. **(C)** Final tumor weights in different treatment groups. **(D)** Body weight changes of mice during treatment. **(E)** Representative H&E staining and immunohistochemical staining of Ki-67, p-EGFR, p-AKT, COX-2, and cleaved-Caspase-3 in xenograft tumor tissues. **(F–J)** Quantitative analysis of p-AKT-positive staining area, p-EGFR-positive staining area, Ki-67-positive staining area, COX-2-positive staining area, and cleaved-Caspase-3-positive staining area. Data are expressed as the mean ± SD. *P < 0.05, **P < 0.01, ***P < 0.001. BBR, berberine; CXB, celecoxib; COX-2, cyclooxygenase-2; H&E, hematoxylin and eosin.

Histological and immunohistochemical analyses further supported the *in vivo* antitumor effect. H&E staining showed more obvious tumor tissue disruption and reduced cellular density in the combination group. IHC staining showed that Ki-67 positivity was reduced after monotherapy and further decreased after BBR + CXB treatment (Figures 8E,H). Consistent with the *in vitro* findings, p-EGFR, p-AKT, and COX-2 staining were markedly decreased in the combination group (Figures 8E–G,I), whereas cleaved-Caspase-3 staining was increased (Figures 8E,J). These results suggest that BBR + CXB exerts enhanced antitumor efficacy in the PC9 xenograft model, accompanied by reduced EGFR/AKT/COX-2-related signaling, decreased tumor cell proliferation, and increased apoptosis *in vivo*.

## 4 Discussion

In this study, we systematically evaluated the combined antitumor effects of BBR and CXB in NSCLC by integrating network pharmacology, molecular docking, Chou-Talalay synergy analysis, *in vitro* functional assays, molecular validation, and an *in vivo* xenograft model. The main findings were that BBR and CXB showed both shared and complementary target profiles in NSCLC; EGFR, AKT1, and PTGS2/COX-2 emerged as candidate hub nodes through PPI topology, GO/KEGG enrichment, and Target-Pathway analysis; and the two compounds exhibited favorable predicted interactions with EGFR, AKT1, and COX-2. Native-ligand re-docking further supported the ability of the docking procedure to reproduce crystallographic ligand poses. More importantly, BBR and CXB synergistically reduced A549 and PC9 cell viability, and the representative working combination dose further suppressed proliferation, colony formation, migration, and invasion while promoting apoptosis. These functional changes were accompanied by reduced EGFR/AKT/COX-2 signaling, downregulation of inflammatory mediators, and changes in apoptosis-related proteins. The PC9 xenograft model further supported the enhanced antitumor effect of the combination *in vivo*.

Combination therapy remains highly relevant in NSCLC because current treatment strategies still face substantial limitations. Although targeted therapy and immune checkpoint blockade have reshaped the therapeutic landscape, durable benefit is restricted by heterogeneous responses, acquired resistance, toxicity, and recurrence. Recent reviews and clinical translational studies emphasize that resistance in NSCLC is driven by diverse mechanisms, including secondary oncogenic alterations, bypass pathway activation, lineage plasticity, immune escape, and tumor microenvironment remodeling (Meyer et al., 2024; Memon et al., 2024; Huang et al., 2025). These challenges support the rationale for exploring combinations that do not rely on a single oncogenic dependency but instead affect multiple biological modules. In this context, the combination of BBR and CXB is biologically plausible: BBR has broad multi-target regulatory properties involving inflammation, oxidative stress, apoptosis, autophagy, metabolism, and cancer-related signaling, whereas CXB mainly acts through the COX-2/PGE_2_ inflammatory axis (Liu et al., 2024; Kuang et al., 2025; Zhou et al., 2025; Xu et al., 2025; Santiso et al., 2024).

The network pharmacology results provide a systems-level framework for this rationale. The identification of 47 shared candidate targets suggests that BBR and CXB may share components of an NSCLC-associated molecular network. Meanwhile, the BBR- and CXB-related complementary target sets indicate that each compound may extend the target coverage beyond the other. This pattern is important because effective drug combinations often depend not only on overlapping targets but also on complementary regulation of disease-relevant modules. In our analysis, AKT1, EGFR, and PTGS2 appeared repeatedly across PPI topological ranking and Target-Pathway analysis, supporting their central positions in the predicted network. Similar integrative strategies combining network pharmacology, molecular docking, and experimental validation have recently been used to study natural products and drug combinations in cancer models (Zhang R. et al., 2019; Boezio et al., 2017; Wang M. et al., 2025; Zhou et al., 2024). However, computational prediction alone is insufficient; its value depends on whether the predicted targets can be linked to measurable biological changes. Therefore, the subsequent *in vitro* and *in vivo* experimental evaluation in this study was essential.

Molecular docking provided additional computational support for potential interactions with EGFR, AKT1, and COX-2, but these results should be interpreted with appropriate caution. BBR, CXB, and the dual-ligand docking systems all showed favorable predicted binding energies, with AKT1-related docking generally exhibiting lower binding energies. Importantly, lower docking energies across different targets should not be interpreted as evidence of greater biological importance or stronger pathway inhibition. Native-ligand re-docking yielded RMSD values below 2.0 Å for all three receptors, supporting acceptable reproduction of the crystallographic ligand poses; however, this validation addresses the docking procedure rather than direct binding of BBR or CXB. Nevertheless, docking mainly reflects spatial compatibility and predicted receptor-ligand affinity under computational conditions. Similarly, the spatial coexistence observed in dual-ligand docking should not be interpreted as evidence of cooperative binding. It cannot independently confirm direct target engagement in living cells or prove pharmacological synergy. In this study, docking should therefore be viewed as a computational link between network prediction and experimental validation. The observed reduction of p-EGFR, p-AKT, and COX-2 after BBR + CXB treatment was consistent with changes involving these candidate targets, but future studies should still confirm direct binding using cellular thermal shift assay, drug affinity responsive target stability assay, surface plasmon resonance, or related target-engagement methods.

The *in vitro* experiments demonstrated that BBR and CXB did not merely produce a stronger combined effect than either monotherapy, but showed quantitative synergy according to Chou–Talalay CI analysis. This finding was further supported by the complementary Bliss, ZIP, HSA, and Loewe models, which yielded positive overall synergy scores in both A549 and PC9 multidose combination matrices. This distinction is important because a lower viability in the combination group alone does not necessarily indicate synergism. The selected dose of 40 μM BBR plus 30 μM CXB was therefore used as a representative working combination dose because it balanced synergistic inhibition with preservation of sufficient viable cells for downstream assays. Excessive cytotoxicity could confound EdU, colony formation, migration, invasion, and apoptosis assays by making it difficult to distinguish specific suppression of malignant phenotypes from nonspecific cell loss. At the representative working combination dose, BBR + CXB consistently inhibited DNA synthesis, clonogenic expansion, wound closure, and invasion, while increasing apoptotic cell populations. These results indicate that the combination affects multiple malignant behaviors of NSCLC cells rather than only reducing metabolic activity. This phenotype is consistent with recent evidence that natural products can regulate apoptosis- and autophagy-related vulnerabilities in NSCLC and may serve as potential adjunctive therapeutic agents (Qin et al., 2024; Zhou et al., 2025; Xu et al., 2025).

Among the molecular changes observed in this study, reduced EGFR and AKT phosphorylation was particularly notable. EGFR activation is a key driver of NSCLC biology, and downstream PI3K/AKT/mTOR signaling contributes to proliferation, survival, metabolic adaptation, invasion, and therapeutic resistance (Huang et al., 2025; Zhang et al., 2025). In our study, BBR + CXB mainly reduced EGFR and AKT phosphorylation rather than markedly decreasing total EGFR or AKT protein expression, consistent with reduced pathway activation. This is biologically meaningful because phosphorylated EGFR and AKT more directly reflect pathway activity. AKT1 also ranked highly in the network and docking analyses, placing it among the candidate molecular nodes warranting further investigation. Therefore, the observed molecular pattern is consistent with attenuation of receptor-linked kinase signaling without substantial reduction in total EGFR or AKT abundance. However, whether these changes are necessary for the antitumor effects of BBR + CXB requires direct pathway-modulation and rescue experiments.

The concurrent downregulation of COX-2 paralleled these kinase-related changes and was accompanied by alterations in inflammatory regulation. COX-2 and its downstream product PGE_2_ are increasingly recognized as important mediators of tumor progression, angiogenesis, immune suppression, and resistance to therapy. Recent lung cancer-specific and general oncology reviews indicate that COX-2 promotes PGE_2_ synthesis and facilitates lung cancer development, while COX-2-targeting strategies may have value in multimodal cancer therapy (Liu et al., 2024; Santiso et al., 2024; Kassab, 2025). In addition, PGE_2_ can shape the tumor microenvironment through EP receptor signaling and suppress antitumor immunity. Recent studies have shown that the COX-2/PGE_2_/EP axis can remodel immune landscapes, limit CD8^+^ T-cell effector expansion, and promote immune escape (Kuang et al., 2025; Santiso et al., 2024; Pelly et al., 2021; Lacher et al., 2024). In the present study, BBR + CXB reduced COX-2 expression and downregulated *PTGS2*, *RELA*/NF-κB, *CXCL8, TNF*, and *IL1B* mRNA expression. These findings suggest that the combination may attenuate a pro-inflammatory tumor cell state alongside its antitumor effects. Although our cell culture and nude mouse models cannot fully assess immune regulation, the decrease in inflammatory mediators provides a rationale for future studies in immune-competent or humanized models.

The apoptosis-related findings further paralleled the observed functional effects. BBR + CXB decreased Bcl-2, increased Bax, reduced the Bcl-2/Bax ratio, and elevated cleaved-Caspase-3 expression. Since AKT activation supports anti-apoptotic signaling and can maintain cancer cell survival, the decrease in p-AKT is consistent with the observed shift toward an apoptosis-associated molecular profile. Thus, these coordinated molecular changes are consistent with a shift from a proliferation- and survival-favorable state toward increased apoptotic susceptibility after combination treatment. This molecular pattern is also consistent with previous studies showing that natural compounds and anti-inflammatory drugs can affect Akt/NFκB/COX2-related pathways in association with antitumor activity (Zhou et al., 2024).

The *in vivo* xenograft results supported the biological relevance of the *in vitro* findings. Compared with either monotherapy, BBR + CXB more strongly inhibited PC9 tumor growth and reduced final tumor weight, without obvious body weight loss under the current experimental conditions. IHC analysis showed reduced Ki-67, p-EGFR, p-AKT, and COX-2 expression, together with increased cleaved-Caspase-3 staining, which was consistent with the molecular changes observed *in vitro*. However, the *in vivo* results should be interpreted as evidence of enhanced combination efficacy rather than definitive proof of *in vivo* synergy. The animal experiment used BBR 10 mg/kg plus CXB 10 mg/kg but did not include total-dose-matched or dose-reduction combination groups. Moreover, the absence of obvious body weight loss alone is insufficient to establish systemic safety. Future studies should therefore test reduced-dose combinations and formal *in vivo* synergy models to determine whether this strategy has a dose-sparing effect, together with more comprehensive toxicological evaluation.

This study has several limitations. First, network pharmacology and molecular docking are predictive methods, and direct target engagement was not experimentally confirmed. Although native-ligand re-docking supported the reproducibility of ligand-pose prediction, it does not establish direct binding of BBR or CXB to EGFR, AKT1, or COX-2 in biological systems. Second, although EGFR/AKT/COX-2 suppression was associated with the observed antitumor phenotypes, pathway activator, knockdown, overexpression, or rescue experiments are needed to determine whether this signaling pattern is causally involved in the combination effect. In addition, the upstream regulatory events responsible for the observed reductions in EGFR and AKT phosphorylation remain unclear and warrant further investigation. Third, only two NSCLC cell lines and one PC9 xenograft model were used; additional cell lines with different genetic backgrounds, including resistant models, patient-derived organoids, and patient-derived xenografts would improve generalizability. Non-malignant human lung cells were also not evaluated, precluding determination of tumor selectivity or a therapeutic index, and direct validation in human NSCLC tissues was not performed. Fourth, the COX-2/PGE_2_ axis is closely associated with immune microenvironment regulation, but nude mouse xenografts cannot fully evaluate immune escape or immune-cell remodeling. In addition, systemic safety evaluation was limited to body weight monitoring, without systematic histological examination of major organs, hematological testing, or serum biochemical assessment. Finally, because BBR has limited solubility and oral bioavailability, pharmacokinetic assessment, formulation optimization, tissue distribution analysis, and systematic safety evaluation will be required before translational development (Xiong et al., 2022; Rauf et al., 2021; Hsu et al., 2024). CXB also requires careful consideration of cardiovascular safety, particularly with prolonged or higher-dose exposure; previous trials have reported dose-related cardiovascular risk at higher doses, whereas the PRECISION trial showed that moderate-dose celecoxib was noninferior to ibuprofen or naproxen for cardiovascular safety (Solomon et al., 2005; Nissen et al., 2016). Therefore, any potential dose-sparing advantage of the BBR–CXB combination remains hypothetical in the absence of formal dose-reduction and therapeutic-window studies, and systematic dose optimization and safety evaluation remain necessary.

## 5 Conclusion

In conclusion, this study demonstrates that BBR and CXB have combined antitumor potential in NSCLC. The combination demonstrated synergistic inhibition of NSCLC cell viability *in vitro*, suppressed malignant phenotypes, promoted apoptosis, and showed enhanced antitumor efficacy in the PC9 xenograft model. At the molecular level, these effects were associated with suppression of EGFR/AKT/COX-2-related signaling, attenuation of inflammatory responses, and changes favoring apoptosis, as summarized in the proposed model in Figure 9. *In vivo*, BBR + CXB further inhibited PC9 xenograft tumor growth without obvious body weight loss. These findings provide experimental evidence and molecular insight for the potential use of BBR and CXB as a network pharmacology-guided combination strategy for NSCLC, although further studies are needed to confirm direct target engagement and optimize translational application.

**FIGURE 9:**
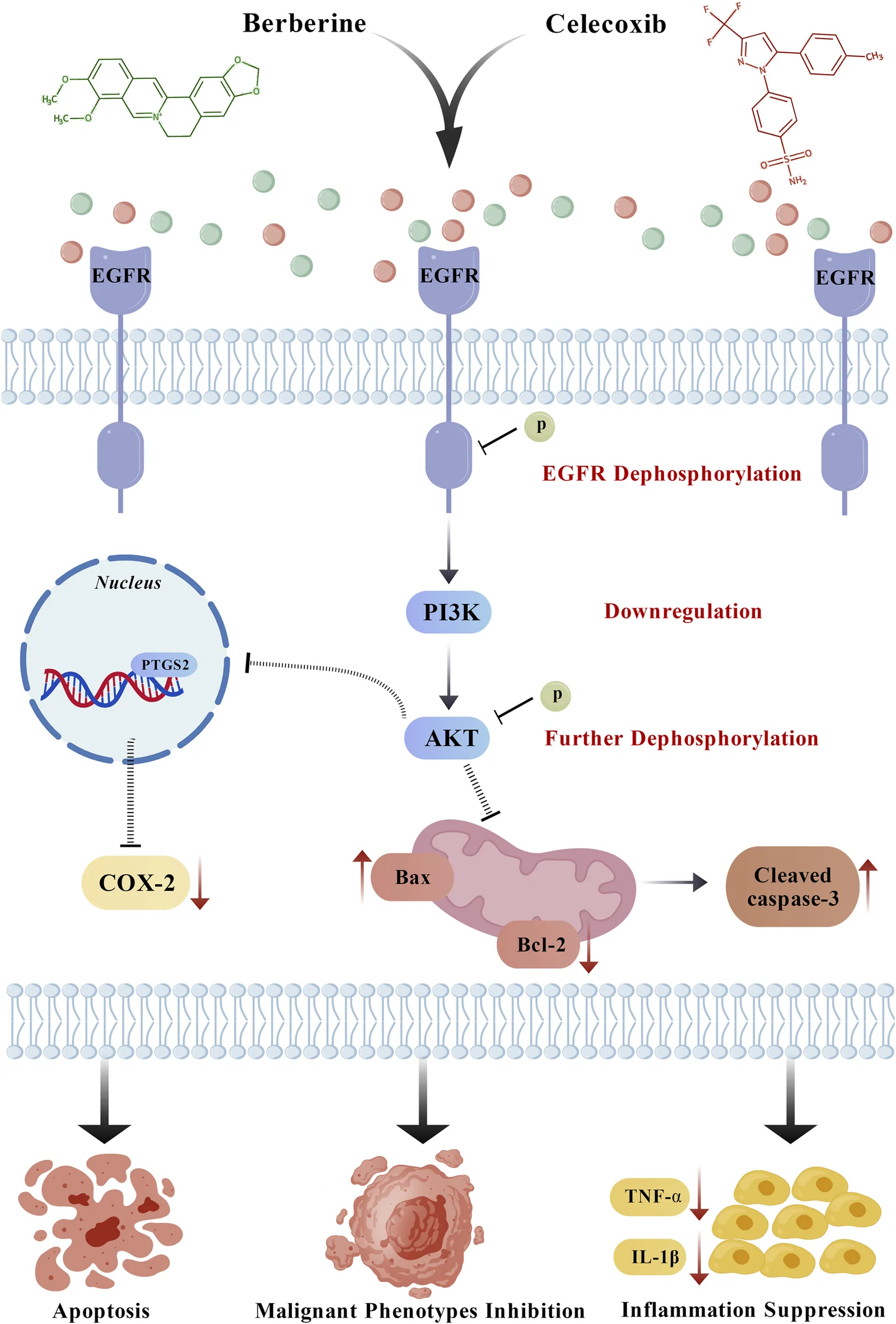
Proposed mechanistic model of BBR + CXB combination treatment against NSCLC. BBR, berberine; CXB, celecoxib; NSCLC, non-small cell lung cancer; COX-2, cyclooxygenase-2.

## Data Availability

The original contributions presented in the study are included in the article and its Supplementary Material, Further inquiries can be directed to the corresponding authors.
