# Supplementary material for: Berberine and celecoxib synergistically suppress non-small cell lung cancer with EGFR/AKT/COX-2-related signaling: network pharmacology, molecular docking and experimental validation
Source: Front Pharmacol. 2026 Sep 29;17:1930125. doi: 10.3389/fphar.2026.1930125 (PMC13658603; doi:10.3389/fphar.2026.1930125)
Supplement: Supplementary file 1 [file Supplementaryfile1.docx]

**Supplementary Figure S1. Native-ligand re-docking of EGFR, COX-2, and AKT1.**
Structural superposition of the crystallographic and re-docked ligand poses for (A) EGFR, (B) COX-2, and (C) AKT1. The original ligand conformations from the crystal structures are shown in **green**, whereas the corresponding re-docked conformations are shown in **orange**. Enlarged views show the overlap between the crystallographic and re-docked poses.

**Supplementary Figure S2. Complementary synergy analyses of BBR and CXB in A549 and PC9 cells using multiple reference models.**
Synergy landscapes of BBR and CXB were generated using SynergyFinder+ based on the multidose combination matrices. (A–C) ZIP, HSA, and Loewe synergy analyses in A549 cells, respectively. (D–F) ZIP, HSA, and Loewe synergy analyses in PC9 cells, respectively. According to the SynergyFinder interpretation guideline, mean synergy scores >10 indicate likely synergistic interactions, scores from −10 to 10 indicate likely additive interactions, and scores <−10 indicate likely antagonistic interactions. The mean synergy score and corresponding P value for each model are shown above the respective plot. BBR, berberine; CXB, celecoxib; ZIP, zero interaction potency; HSA, highest single agent.
