# Supplementary material for: Berberine and celecoxib synergistically suppress non-small cell lung cancer with EGFR/AKT/COX-2-related signaling: network pharmacology, molecular docking and experimental validation
Source: Front Pharmacol. 2026 Sep 29;17:1930125. doi: 10.3389/fphar.2026.1930125 (PMC13658603; doi:10.3389/fphar.2026.1930125)
Supplement: Supplementary file 2 [file Table1.docx]

Supplementary Table S1. Antibodies used for western blotting and immunohistochemistry

| **Target** |  | **Antibody** | **Application** | **Supplier** | **Cat. No.** | **Dilution** |
| --- | --- | --- | --- | --- | --- | --- |
| EGFR |  | anti-EGFR antibody | WB | CST | #4267 | 1:1000 |
| p-EGFR |  | anti-phospho-EGFR (Tyr1068) antibody | WB | CST | #3777 | 1:1000 |
| p-EGFR |  | anti-phospho-EGFR (Tyr1068) antibody | IHC | CST | #3777 | 1:100 |
| AKT |  | anti-AKT antibody | WB | CST | #9272 | 1:1000 |
| p-AKT |  | anti-phospho-AKT (Ser473) antibody | WB | CST | #4060 | 1:2000 |
| p-AKT |  | anti-phospho-AKT (Ser473) antibody | IHC | CST | #4060 | 1:100 |
| COX-2 |  | anti-COX-2 antibody | WB | Abcam | ab15191 | 1 μg/mL |
| COX-2 |  | anti-COX-2 antibody | IHC | Abcam | ab15191 | 1:200 |
| TNF-α |  | anti-TNF-α antibody | WB | CST | #6945 | 1:1000 |
| IL-1β |  | anti-IL-1β antibody | WB | Abcam | ab216995 | 1:1000 |
| Bax |  | anti-Bax antibody | WB | CST | #2772 | 1:1000 |
| Bcl-2 |  | anti-Bcl-2 antibody | WB | CST | #2876 | 1:1000 |
| cleaved-Caspase-3 |  | anti-cleaved-Caspase-3 (Asp175) antibody | WB | CST | #9664 | 1:1000 |
| cleaved-Caspase-3 |  | anti-cleaved-Caspase-3 (Asp175) antibody | IHC | CST | #9664 | 1:100 |
| GAPDH |  | anti-GAPDH antibody | WB | CST | #5174 | 1:1000 |
| Ki-67 |  | anti-Ki-67 antibody | IHC | Abcam | ab15580 | 1:200 |
| Rabbit IgG |  | HRP-conjugated goat anti-rabbit IgG H&L secondary antibody | WB | Abcam | ab205718 | 1:5000 |
| Rabbit IgG |  | HRP-conjugated goat anti-rabbit IgG H&L secondary antibody | IHC | Abcam | ab205718 | 1:20000 |

Supplementary Table S2. Primer sequences used for qRT-PCR

| **Primer** | **Sequence** |
| --- | --- |
| GAPDH | F: 5'-GTCTCCTCTGACTTCAACAGCG-3′ |
|  | R: 5′-ACCACCCTGTTGCTGTAGCCAA-3′ |
| EGFR | F: 5′-AACACCCTGGTCTGGAAGTACG-3′ |
|  | R: 5′-TCGTTGGACAGCCTTCAAGACC-3′ |
| AKT1 | F: 5′-TGGACTACCTGCACTCGGAGAA-3′ |
|  | R: 5′-GTGCCGCAAAAGGTCTTCATGG-3′ |
| PTGS2 | F: 5′-CGGTGAAACTCTGGCTAGACAG-3′ |
|  | R: 5′-GCAAACCGTAGATGCTCAGGGA-3′ |
| NF-κB | F: 5′-TGAACCGAAACTCTGGCAGCTG-3′ |
|  | R: 5′-CATCAGCTTGCGAAAAGGAGCC-3′ |
| CXCL8 | F: 5′-GAGAGTGATTGAGAGTGGACCAC-3′ |
|  | R: 5′-CACAACCCTCTGCACCCAGTTT-3′ |
| TNF | F: 5′-CTCTTCTGCCTGCTGCACTTTG-3′ |
|  | R: 5′-ATGGGCTACAGGCTTGTCACTC-3′ |
| IL-1B | F: 5′-CCACAGACCTTCCAGGAGAATG-3′ |
|  | R: 5′-GTGCAGTTCAGTGATCGTACAGG-3′ |

Supplementary Table S3. Shared and complementary candidate targets identified by Venn intersection analysis among BBR-related targets, CXB-related targets, and NSCLC-associated targets.

| 47 | 29 | 17 |
| --- | --- | --- |
| BOTH | B+N-S | C+N-S |
| PPARD | CXCL8 | MPO |
| CYP2E1 | CDC42 | HDAC3 |
| CYP2A6 | RAC1 | ABCC3 |
| CXCR1 | CYP1B1 | ABCC4 |
| PTPRC | AKR1C3 | ABCC2 |
| RXRA | TERT | SLC29A1 |
| STAT3 | CYP1A1 | EDNRB |
| CXCR2 | NFKB1 | PDPK1 |
| MAPK3 | KDM1A | AGTR1 |
| CCR2 | HIF1A | ALOX5 |
| FYN | PPARA | HDAC2 |
| CCR5 | IDO1 | HDAC1 |
| EDNRA | P2RX7 | PGR |
| ACHE | PTPN1 | CA9 |
| PRKCA | APP | PPARG |
| FLT1 | RUNX1 | SMAD3 |
| MMP9 | GSK3B | NEU1 |
| ESR2 | ABCB1 |  |
| CASP1 | FEN1 |  |
| NR3C1 | MAPT |  |
| ERBB2 | ALDH1A1 |  |
| MMP1 | MEN1 |  |
| ELANE | BIRC5 |  |
| PTGS1 | NOS2 |  |
| AR | NOS3 |  |
| ADRB2 | HSP90AB1 |  |
| LCK | PRKACA |  |
| TSHR | PRSS1 |  |
| PTGS2 | NCOA2 |  |
| MAPK14 |  |  |
| ESR1 |  |  |
| HDAC6 |  |  |
| CYP1A2 |  |  |
| KCNH2 |  |  |
| CYP2D6 |  |  |
| CYP3A4 |  |  |
| EGFR |  |  |
| MAPK1 |  |  |
| LMNA |  |  |
| IDH1 |  |  |
| NFE2L2 |  |  |
| RELA |  |  |
| RAF1 |  |  |
| AKT1 |  |  |
| PCK1 |  |  |
| FOXO3 |  |  |
| BRCA1 |  |  |

Supplementary Table S4. Top 10 hub targets ranked by PPI network topology and Target–Pathway network analysis.

| **Clossness unDir** | | **Betweenness unDir** | | **Degree unDir** | | **Target-Pathway** | |
| --- | --- | --- | --- | --- | --- | --- | --- |
| AKT1 | 0.009009009 | AKT1 | 636.2342509 | AKT1 | 73 | MAPK1 | 20 |
| STAT3 | 0.008264463 | ESR1 | 424.5260579 | PTGS2 | 63 | MAPK3 | 20 |
| NFKB1 | 0.008196721 | MMP9 | 414.0101345 | NFKB1 | 62 | NFKB1 | 18 |
| EGFR | 0.008130081 | ABCB1 | 387.5258678 | EGFR | 61 | AKT1 | 17 |
| ESR1 | 0.008064516 | PPARG | 350.7786975 | ESR1 | 60 | RELA | 17 |
| HIF1A | 0.007874016 | STAT3 | 304.2345928 | HIF1A | 57 | EGFR | 14 |
| PPARG | 0.007751938 | EGFR | 295.8002648 | PPARG | 56 | PTGS2 | 12 |
| GSK3B | 0.007692308 | PTGS2 | 278.3510528 | MMP9 | 54 | GSK3B | 10 |
| MAPK3 | 0.007633588 | HSP90AB1 | 262.4509163 | MAPK3 | 53 | CXCL8 | 8 |
| PTGS2 | 0.007518797 | HIF1A | 258.0992155 | CXCL8 | 52 | MMP9 | 8 |

Supplementary Table S5. Native-ligand re-docking validation of the molecular docking protocol.

| **Target** | **PDB ID** | **Native ligand** | **Re-docking binding energy (kcal/mol)** | **RMSD (Å)** | **Interpretation** |
| --- | --- | --- | --- | --- | --- |
| EGFR | 1YY9 | NAG | -5.768 | 0.319 | Acceptable |
| AKT1 | 5KCV | Miransertib (ARQ 092) | -12.92 | 0.581 | Acceptable |
| COX-2 | 5F19 | NAG | -5.102 | 0.647 | Acceptable |

Re-docking results for EGFR, AKT1, and COX-2 are summarized with the corresponding PDB IDs, native ligands, predicted binding energies, and root-mean-square deviation (RMSD) values between the crystallographic and re-docked ligand poses. An RMSD value < 2.0 Å was considered indicative of acceptable reproduction of the crystallographic binding conformation.

Supplementary Table S6. Multimodel synergy analysis of BBR and CXB in A549 and PC9 cells using SynergyFinder+.

| **Cell line** | **Synergy model** | **Mean synergy score** | **P value** |
| --- | --- | --- | --- |
| A549 | ZIP | 14.53 | 5.88 × 10⁻⁹ |
| A549 | HSA | 27.31 | 6.27 × 10⁻⁸ |
| A549 | Loewe | 17.37 | 9.40 × 10⁻⁵ |
| A549 | Bliss | 14.61 | 1.30 × 10⁻⁸ |
| PC9 | ZIP | 13.08 | 1.34 × 10⁻⁷ |
| PC9 | HSA | 28.06 | 1.62 × 10⁻⁷ |
| PC9 | Loewe | 17.28 | 5.12 × 10⁻⁵ |
| PC9 | Bliss | 13.04 | 4.72 × 10⁻⁷ |

The multidose BBR–CXB combination matrices were analyzed using the ZIP, HSA, Loewe, and Bliss reference models in SynergyFinder+. Mean synergy scores and the corresponding P values are presented for each model and cell line. Positive synergy scores indicate that the observed combination effect exceeded the expected effect under the corresponding reference model. BBR, berberine; CXB, celecoxib; ZIP, zero interaction potency; HSA, highest single agent.
